# Roostocks/Scion/Nitrogen Interactions Affect Secondary Metabolism in the Grape Berry

**DOI:** 10.3389/fpls.2016.01134

**Published:** 2016-08-09

**Authors:** Aude Habran, Mauro Commisso, Pierre Helwi, Ghislaine Hilbert, Stefano Negri, Nathalie Ollat, Eric Gomès, Cornelis van Leeuwen, Flavia Guzzo, Serge Delrot

**Affiliations:** ^1^UMR 1287, EGFV, Bordeaux Sciences Agro, Institt National de la Recherche Agronomique, Université de BordeauxVillenave d'Ornon, France; ^2^Biotechnology Department, University of VeronaVerona, Italy

**Keywords:** grapevine, berry, rootstock, nitrogen, metabolomics

## Abstract

The present work investigates the interactions between soil content, rootstock, and scion by focusing on the effects of roostocks and nitrogen supply on grape berry content. Scions of Cabernet Sauvignon (CS) and Pinot Noir (PN) varieties were grafted either on Riparia Gloire de Montpellier (RGM) or 110 Richter (110R) rootstock. The 4 rooststock/scion combinations were fertilized with 3 different levels of nitrogen after fruit set. Both in 2013 and 2014, N supply increased N uptake by the plants, and N content both in vegetative and reproductory organs. Rootstock, variety and year affected berry weight at harvest, while nitrogen did not affect significantly this parameter. Grafting on RGM consistently increased berry weight compared to 110R. PN consistently produced bigger berries than CS. CS berries were heavier in 2014 than in 2013, but the year effect was less marked for PN berries. The berries were collected between veraison and maturity, separated in skin and pulp, and their content was analyzed by conventional analytical procedures and untargeted metabolomics. For anthocyanins, the relative quantitation was fairly comparable with both LC-MS determination and HPLC-DAD, which is a fully quantitative technique. The data show complex responses of the metabolite content (sugars, organic acids, amino acids, anthocyanins, flavonols, flavan-3-ols/procyanidins, stilbenes, hydroxycinnamic, and hydroxybenzoic acids) that depend on the rootstock, the scion, the vintage, the nitrogen level, the berry compartment. This opens a wide range of possibilities to adjust the content of these compounds through the choice of the roostock, variety and nitrogen fertilization.

## Introduction

Since the early Twentieth-Century, most vineyards over the world (with the exceptions of Argentina, Australia, Chile, China) are grafted onto a rootstock of either a single American *Vitis* species or hybrids between *V. berlandieri, V. riparia, V. rupestris* (Whiting, 2004; Ollat et al., 2015). In addition to phylloxera, rootstocks contribute to the control of other soil-borne pests. They may also allow to withstand climate or adverse soil conditions such as drought, or high salt or lime content (Galet and Smith, 1998; Whiting et al., 2005), and to cope with problems of mineral nutrition (Keller et al., 2001; Lecourt et al., 2015). The rootstocks indirectly modify whole plant development by affecting the vigor of the scion (Tandonnet et al., 2010), biomass accumulation and distribution (Paranychianakis et al., 2004; Smart et al., 2006; Koundouras et al., 2008; Alsina et al., 2011), yield (Main et al., 2002; Jones et al., 2009), and phenology (Pongracz, 1983; Whiting et al., 2005). They are directly involved in water and ion uptake from the soil and their translocation to the upper part of the plant. It is therefore important to understand the interactions between soil content, rootstocks and scions.

Among the nutrients present in the soil solution, nitrogen is the most important for the control of vigor, yield, and berry quality. Increasing constraints are put on the use of nitrogen fertilization in order to avoid the pollution of ground water table. Even though grapevine requests smaller amounts of nitrogen than most other crops (30 kg/ha vs. 100–200 kg/ha), the ban of nitrogen fertilization on slopes steeper than 15% and its limitation in other conditions may be a problem in viticulture. This is particularly the case when intercropping is used, because the cover crop generally competes with the vines for nitrogen (Celette et al., 2009). Furthermore, the price of nitrogen fertilizers will increase due to the energy cost of their chemical synthesis.

In grapevine, assimilation of nitrogen may occur in the roots, trunk, stem, leaves and berries (Wermelinger et al., 1991). Therefore, various forms of nitrogen (nitrate, ammonium, amino acids, small peptides, proteins) may be found in these organs. Nitrogen is principally stored under the form of arginine (Nassar and Kliewer, 1966; Kliewer et al., 1967) and may be remobilized from the trunk and the roots toward the leaves, the stems and the fruits. This remobilization which occurs between bud burst and ripening depends on the reserves made during the previous year.

Premium wines are often obtained with grapevines that are grown on poor and superficial soils, and sometimes on slopes (Delas, 2000). Mild water deficit and moderate nitrogen availability direct berry metabolism toward the synthesis of phenolic and aromatic compounds. Excess nitrogen results in high vigor and increased *Botrytis cinerea* infection, which is detrimental to wine quality (Choné et al., 2001). Hence, the grape grower has to manage nitrogen supply in such a way that vegetative and reproductive growth is sufficient while nitrogen deficiency is avoided.

The present work investigates the interactions between soil content, rootstock and scion by focusing on the effects of roostocks and nitrogen supply on berry content for two scion varieties. Although some work has addressed the effects of nitrogen supply and rootstock effect on berry composition and wine quality, their combined action has only recently started to be investigated (Lecourt et al., 2015). In particular, non-targeted metabolite profiling has not yet been used to investigate these relationships in detail.

## Materials and methods

### Plant material

Grapevines [*V. vinifera* cv. Cabernet-Sauvignon (CS) clone 169, and Pinot noir (PN) clone 777] grafted on either RGM clone 1 or 110R clone 152 rootstocks were used. The two varieties were chosen because of their contrasting profiles in secondary metabolites. RGM and 110R are rootstocks conferring, respectively, low and high vigor to the scion (Tandonnet et al., 2010). 128 plants corresponding to the four rootstock/scion combinations (110R/ CS; 110R/PN; RGM/CS; RGM/PN) were grafted in 2011. 32 plants/combination were planted in 10 L plastic pots containing loam, perlite and sand (4:3:3, v:v:v) and cultivated outside for the experiment. Vines were pruned with 2 spurs of 2 buds (4 buds per vine). Water and nutrients were supplied 3 times per day (1.2 L/day/plant) by drip irrigation with complete nutrient solutions. In 2013 and 2014, from budbreak to fruit set, all plants were supplied with a solution containing 1.4 mM nitrogen. After fruit set, 3 different concentrations of nitrogen were used for the fertirrigation: 0.8 mM N, 1.4 mM N, and 3.6 mM N (denominated N–, N0 and N+, respectively). Based on previous work (Hilbert et al., 2003; Lecourt et al., 2015), N–, N0 and N+ are considered as limited, mean and excessive nitrogen levels. Nitrogen was supplied as potassium nitrate, ammonium phosphate, calcium nitrate, ammonium sulfate and sequestrene. Except for nitrogen, all solution had the same non-limiting concentrations of other mineral elements. Leaf area was determined as described by Mabrouk et al. (1997). Ten plants per combination and per treatment were randomly distributed in the experiment.

### Samples collection

Three groups of three plants of each combination (rootstock/scion) were constituted to obtain three biological replicates. Berries were sampled during the 2013 and 2014 growing season at three time points, veraison (V), mid-maturity (MM, 30 days after mid-veraison), and maturity (M, 48 days after mid-veraison). Each biological replicate comprised twenty five berries randomly sampled at different anatomical and exposure positions of the clusters. All clusters were equally exposed to light. Berries were immediately frozen in liquid nitrogen and stored at −80°C. For the analysis, the skins were separated from the pulp and the seeds, frozen at −80°C and freeze-dried. The dried skins and pulp were powdered.

### Plant nitrogen (N) status and berry nitrogen content

The leaf blades and the petiole total nitrogen content were determined according to the Dumas method with an elemental auto-analyzer (Flash EA 1112 series, Thermo Fisher Scientific, Courtaboeuf, France). In parallel, berry nitrogen content was assessed by Yeast Available Nitrogen (YAN) in grape juice at harvest. One hundred berries from each replicate were sampled and pressed. The juice was analyzed with a Fourier Transform Infra-Red spectrometer (FTIR, WineScan FOSS®, FRANCE, 92000 Nanterre).

### Analysis of primary metabolites

#### Sugar and organic acid analysis

An aliquot of 80 mg dry powder of samples (pulp and skin) was extracted, and sugar and organic acids were analyzed according to Bobeica et al. (2015).

#### Amino acid content

Amino acid concentration in berries was analyzed according to Pereira et al. (2006) with modifications. After derivatization with AccQTag Ultra derivatization reagent (Waters, Milford, MA, USA), amino acids were analyzed using an UltiMate 3000 UHPLC system equipped with FLD-3000 Fluorescence Detector (Thermo Electron SAS, Waltham, MA USA). Separation was performed on a AccQ•Tag Ultra column, 2,1 × 100 mm, 1, 7 μm (Waters, Milford, MA, USA) at 37°C with elution at 0.5 ml min^−1^ according to the following gradient (v/v): 0 min 93% A 4.2% B 2.8% C, 6.5 min 95% A 8.4% B 5.6% C, 9 min 78% A 13.2% B 8.8% C, 11 min 71% A 17.4%B 11.6% C linear for 2 min, 14 min 60% B 40% C linear for 1 min, 15 min 93% A 4.2% B 2.8% C (eluent A, sodium acetate buffer, 140 mM at pH 5.7; eluent B, acetonitrile; eluent C, water). Chromatograms corresponding to excitation at 250 nm and emission at 395 nm were recorded. The compounds were quantified by their peak area with Chromeleon software, version 7.1 (Thermo Electron SAS, Waltham, MA, USA) using external standards. Chemical standards were purchased from Sigma (St Louis, MO, USA). Twenty amino acids were identified and quantified as described by Pereira et al. (2006). The results were expressed in nmoles/g DW.

### Untargeted metabolomic analyses

Powdered skins were extracted with forty volumes of ice cold methanol containing 10% of water and 0.1% formic acid; powdered pulps were extracted with ten volumes of ice cold methanol containing 10% of water. Extracts were sonicated at 40 kHz for 20 min in an ultrasonic bath (Falc Instruments, Bergamo, Italy) at room temperature, centrifuged for 15 min at 16,000 g at 4°C and finally stored at −20°C or immediately diluted for LC-MS (Liquid chromatography-mass spectrometry) and LC-DAD (Liquid chromatography-diode array detector) analyses. In detail, skin and pulp extracts were diluted 1:2 with water LC-MS grade for LC-MS, and respectively, 1:4 and 2:3 for LC-DAD. Finally, the solutions were filtered through 0.2 μm pore filters prior the injection.

The chromatographic analyses were carried out with two Beckman Coulter Gold 127 HPLC system (Beckman Coulter, Fullerton, CA) equipped with a C18 guard column (7.5 × 2.1 mm) and an analytical Alltima HP C18 column (150 × 2.1 mm, particule size 3 μm; Alltech Associates Inc, Derfield, IL), linked with either a Bruker Esquire 6000 mass spectrometer or a System Gold 168 Diode Array Detector (Beckman Coulter). The HPLC system was also on-line with a Beckman Coulter System Gold 508 autosampler in which samples were maintained at 4°C. Two solvents were used: 5% (v/v) acetonitrile, 0.5% (v/v) formic acid in water (solvent A) and 100% acetonitrile (solvent B). A solvent gradient was established from 0 to 10% B in 2 min, from 10 to 20% B in 10 min, from 20 to 25% B in 2 min, from 25 to 70% B in 7 min, isocratic flow for 5 min and from 70 to 90% in 1 min, and finally from 90 to 0% in 1 min. Then, the column was equilibrated for 20 min in 100% of solvent A. The injection volume was 20 μL for all samples.

The Bruker ion trap Esquire 6000 mass spectrometer was equipped with electrospray ionization source (ESI). The analyses were performed both in positive and negative modes, setting the scan among 50–3000 m/z and a target mass of 400 m/z. The ESI values were 50 psi and 350°C for the nitrogen nebulizing gas and 10 L/min for the drying gas. Mass spectra were recorded using an Averages of 5 spectra and Max Accu Time of 100 ms. The fragmentation was carried out in AutoMS, fragmenting molecules up to three times. Helium was injected to induce molecule fragmentation. MS data were collected with Bruker Daltonics Esquire 5.2 Control software and processed with Esquire 3.2 Data Analysis software (Bruker Daltonik GmbH, Bremen, Germany). MS data files were converted from .d extension to net.cdf and submitted to mzMine 2.10 (http://mzmine.sourceforge.net). The resulting data matrix, reporting the samples and the peak areas of the detected signals, was imported in Simca 13 (Umetrix, Sweden) software to perform the statistical analysis. Metabolite identification was performed by comparing the retention times, m/z and fragmentation patterns of a signal with those of authentic commercial standards included in our home-made library. When no match was observed, the m/z and the fragmentation pattern of the putative molecule were compared with those reported in literature or in on-line databases (massbank.jp; hmbd.ca).

The absorbances were recorded among 190–600 nm (UV-Vis) in LC-DAD analyses. Molecules identified as anthocyanins and hydroxycinnamic acid derivatives in LC-MS were confirmed by measuring their absorbance at 520 and 320 nm, respectively. For anthocyanin quantification, authentic commercial standard Kuromanin chloride (Sigma Aldrich) was earlier dissolved in methanol (Sigma Aldrich), diluted 1:2 with water and 20 μL were injected to the LC-DAD system. 0.01, 0.025, 0.05, 0.075, 0.1, 0.25, 0.5, 0.75, 1, 5, and 10 μg of standard were analyzed in triplicate and the peak areas at 520 nm were annotated. The final equation (*R*^2^:0.9994) was used to assess the amount of the different grape anthocyanins as mg of Kuromanin's equivalents in 100 g of powder.

### Statistical analyses

The MS data matrix was imported into Simca 13 software (Umetrix, Sweden) and unsupervised Principal Component (PCA) and supervised Orthogonal Partial Least Square Discriminant (OPLS-DA) analyses were performed using centering and pareto scaling. The unsupervised PCA was carried out to observe homogeneous sample clusters that were used as Y classes in the supervised OPLS-DA. As final output, molecules responsible for cluster separation in OPLS-DA were identified by plotting the pq(corr), i.e. the correlation between p (based on the X component, the metabolites) and q (based on the Y component, the classes), against p. The statistical analyses were validated by performing: (a) permutation test (200 permutations); (b) CV-ANOVA (*p* < 0.05); (c) *t*-test (*p* < 0.05) for a cluster characterizing molecule.

## Results

### Plant vigor

#### Plant vigor was estimated by measurement of pruning weight, leaf surface area, and berry yield

Pruning weights were higher in 2014 than in 2013 (Table 1). The pruning weight of CS plants depended on the rootstock genotype and tended to be higher when the scions were grafted on RGM than on 110R, while the rootstock did not affect the pruning weight for PN. In 2013, the pruning weight was increased by N supply, whatever the rootstock:scion combination, while this was not the case in 2014.

**Table 1:** **Pruning wood weight as affected by nitrogen supply**.

**Years**	**Scion/rootstock combination**	**Nitrate supply**	**Wood weight (g)**	**YAN (mg N/L)**	**Analysis of variance**	**Wood weight (g)**	**YAN (mg N/L)**
2013	CS/RGM	N–	187.50 ± 34.18	120.00±	Years (Y)	^***^	–
		N+	242.50 ± 32.68	241.00±	Treatment (T)	^*^	^***^
	CS/110R	N–	164.50 ± 20.20	180.00±	Variety (V)	^***^	^*^
		N+	208.00 ± 14.94	252.00±	Rootstock (R)	^**^	NS
	PN/RGM	N–	155.00 ± 21.47	180.00±	Y/T	^**^	–
		N+	190.50 ± 10.92	215.00±	Y/V	^***^	–
	PN/110R	N–	158.00 ± 15.31	164.00±	V/R	^*^	NS
		N+	183.50 ± 11.07	223.00±	T/V	NS	NS
2014	CS/RGM	N–	298.00 ± 66.22	252.67 ± 45.79	Y/R	NS	–
		N+	255.50 ± 60.23	410.33 ± 11.68	T/R	NS	NS
	CS/110R	N–	266.00 ± 60.45	295.67 ± 30.83	Y/T/V	NS	–
		N+	266.11 ± 94.30	490.00 ± 26.00	Y/T/R	NS	–
	PN/RGM	N–	179.00 ± 28.07	268.00 ± 07.21	Y/V/R	NS	–
		N+	217.00 ± 26.10	518.00 ± 25.87	T/V/R	NS	NS
	PN/110R	N–	203.00 ± 44.11	260.33 ± 47.35	Y/T/V/R	NS	–
		N+	197.50 ± 50.84	568.00 ± 47.29			

Values are means of 3 independent replicates + SE. N−: 0.8 mM N; N+: 3.6 mM N. One factor (N treatment) Anova tests were made, as well as Tukey tests. For each rootstock/variety combination, a and b indicate significantly different values between N– and N+ treatment. ^±^Unique value. Statistical analyses were done using an analysis of variance with years (Y), rootstock (R), treatment (T), variety (V), and their interaction effects (ns, P > 0.05;

* P < 0.05;

** P < 0.01;

*** P < 0.001).

The rootstock genotype did not affect leaf area and nitrogen fertilization only increased the area of secondary leaves (Supplementary Table 1). Both the variety and the year significantly affected leaf area and interacted together.

While rootstock, variety and year affected berry weight at harvest, nitrogen did not have significant effect (Supplementary Table 2). Both in 2013 and 2014, grafting on RGM resulted in higher berry weight than grafting on 110R. PN consistently produced bigger berries than CS. CS berries were heavier in 2014 than in 2013, but the year effect was less marked for PN berries.

### Nitrogen uptake

Nitrogen uptake was assessed by measuring the N content of leaves (Table 2), petioles (Supplementary Table 3) and yeast assimilable nitrogen in the must (Supplementary Table 4). All these parameters concur to indicate that both in 2013 and 2014, N supply increased N uptake by the plants, and N content both in vegetative and reproductive organs. This is further supported by the amino acid analysis presented below (Table 3). The responses observed to N treatment did not significantly differ among the different rootstock/scion combinations tested.

**Table 2:** **Leaf total N content as affected by nitrogen supply**.

**Years**	**Scion/rootstock combination**	**Nitrate supply**	**Leaf nitrogen nitrogen Content (%)**	**Petiole nitrogen content (%)**	**Analysis of variance**	**Leaf nitrogen content (%)**	**Petiole nitrogen content (%)**
2013	CS/RGM	N–	1.00 ± 0.10a	0.23 ± 0.01	Years (Y)	^**^	NS
		N+	1.77 ± 0.41b	0.37 ± 0.12	Treatment (T)	^***^	^*^
	CS/110R	N–	1.14 ± 0.17a	0.25 ± 0.05	Variety (V)	^**^	NS
		N+	1.79 ± 0.30b	0.48 ± 0.18	Rootstock (R)	NS	NS
	PN/RGM	N–	1.10±	0.28±	Y/T	NS	NS
		N+	1.76±	0.37±	Y/V	NS	NS
	PN/110R	N–	1.12±	0.21±	V/R	NS	NS
		N+	1.87±	0.37±	T/V	NS	NS
2014	CS/RGM	N–	1.10 ± 0.08a	0.38 ± 0.10	Y/R	NS	NS
		N+	2.10 ± 0.09b	0.66 ± 0.10	T/R	NS	NS
	CS/110R	N–	1.17 ± 0.20a	0.39 ± 0.04	Y/T/V	NS	NS
		N+	2.02 ± 0.07b	0.55 ± 0.12	Y/T/R	NS	NS
	PN/RGM	N–	1.43 ± 0.08a	0.33 ± 0.01	Y/V/R	NS	NS
		N+	2.22 ± 0.09b	0.63 ± 0.12	T/V/R	NS	NS
	PN/110R	N–	1.36 ± 0.04a	0.30 ± 0.02	Y/T/V/R	NS	NS
		N+	2.43 ± 0.02b	0.60 ± 0.08			

^±^Unique value. Values are means of 3 independent replicates + SE. N−: 0.8 mM N; N+: 3.6 mM N. One factor (N treatment) Anova tests were made, as well as Tukey tests. For each rootstock/variety combination, a and b indicate significantly different values between N− and N+ treatment. ^±^Unique value. Statistical analyses were done using an analysis of variance with years (Y), rootstock (R), treatment (T), variety (V), and their interaction effects (ns, P > 0.05;

* P < 0.05;

** P < 0.01;

*** P < 0.001).

Table 3**Effect of nitrogen supply on the primary metabolites of berries for four rootstock/scion combinations**.

**Table:** 

**(A)**								
**Year**	**Scion/rootstock combination**		**Nitrate supply**	**Malate (mmoles/g DW)**	**Tartrate (mmoles/g DW)**	**Glucose (mmoles/g DW)**	**Fructose (mmoles/g DW)**	**Total AA (nmol/mg DW)**
2013	CS/RGM	Skin	N−	3.09 ± 0.31	6.45 ± 0.68	33.00 ± 2.97	19.27 ± 2.99	32.01 ± 7.32
			N+	3.93 ± 0.29^*^	5.92 ± 0.68	31.08 ± 1.47	20.29 ± 1.18	71.02 ± 19.63^*^
		Pulp	N−	3.84 ± 0.36	5.25 ± 0.30	119.62 ± 31.21	98.56 ± 25.88	22.76 ± 6.73
			N+	4.93 ± 0.92	5.88 ± 0.88	110.34 ± 18.52	94.90 ± 13.42	84.95 ± 31.31
	CS/110R	Skin	N−	3.37 ± 0.21	5.81 ± 0.81	37.63 ± 0.26	24.10 ± 0.75	20.64 ± 0.67
			N+	4.67 ± 0.50^*^	5.42 ± 0.23	32.24 ± 3.55	24.57 ± 5.48	125.57 ± 42.34^*^
		Pulp	N−	3.14 ± 0.39	3.56 ± 0.90	93.59 ± 27.37	84.53 ± 23.18	42.72 ± 48.09
			N+	5.26 ± 0.77^*^	5.60 ± 0.82^*^	124.49 ± 49.31	107.17 ± 38.64	74.81 ± 20.18
	PN/RGM	Skin	N−	3.19 ± 0.36	5.43 ± 0.26	33.35 ± 18.48	33.35 ± 15.02	18.86 ± 3.84
			N+	3.41 ± 0.24	6.54 ± 0.28^**^	18.98 ± 1.87	22.52 ± 1.92	43.48 ± 6.62^**^
		Pulp	N−	2.13 ± 0.26	3.70 ± 0.08	77.87 ± 12.52	72.97 ± 12.74	25.85 ± 0.69
			N+	2.50 ± 0.02	4.32 ± 0.13^**^	72.02 ± 18.98	67.59 ± 15.75	24.48 ± 12.27
	PN/110R	Skin	N−	3.72 ± 0.17	6.05 ± 0.15	21.00 ± 4.02	24.36 ± 6.38	12.79 ± 1.75
			N+	4.23 ± 0.28	5.90 ± 0.12	21.95 ± 1.92	26.81 ± 2.29	23.14 ± 8.02
		Pulp	N−	2.74 ± 0.33	4.24 ± 0.18	92.48 ± 8.43	83.93 ± 4.04	31.4 ± 9.08
			N+	3.18 ± 0.15	4.54 ± 0.19	67.19 ± 4.15^*^	64.16 ± 1.61^**^	29.48 ± 13.9
2014	CS/RGM	Skin	N−	2.65 ± 0.19	6.10 ± 0.09	34.15 ± 3.16	22.00 ± 1.74	64.77 ± 25.11
			N+	3.29 ± 0.66	6.39 ± 0.06^*^	27.95 ± 4.14	20.76 ± 2.77	128.5 ± 3.10^*^
		Pulp	N−	4.03 ± 1.56	6.05 ± 1.48	128.35 ± 34.35	115.51 ± 28.93	77.12 ± 33.39
			N+	3.45 ± 0.11	4.43 ± 0.14	79.14 ± 9.24	70.94 ± 6.15	105.31 ± 12.01
	CS/110R	Skin	N−	3.10 ± 0.49	5.60 ± 0.45	40.83 ± 1.91	26.78 ± 0.71	90.25 ± 31.59
			N+	3.84 ± 0.69	5.78 ± 0.70	33.66 ± 0.52^*^	27.76 ± 1.29	151.33 ± 18.64
		Pulp	N−	3.07 ± 0.49	4.75 ± 0.45	133.57 ± 38.13	113.69 ± 28.86	70.08 ± 32.88
			N+	2.89 ± 0.17	3.63 ± 0.83	87.88 ± 23.69	82.02 ± 19.79	83.19 ± 18.90
	PN/RGM	Skin	N−	3.06 ± 0.60	5.71 ± 0.51	26.57 ± 11.11	24.84 ± 10.32	45.19 ± 37.1
			N+	5.27 ± 0.48^**^	6.22 ± 0.26	24.95 ± 2.12	26.56 ± 2.35	58.74 ± 9.4
		Pulp	N−	3.24 ± 0.23	3.83 ± 0.41	79.32 ± 9.38	67.87 ± 7.00	55 ± 2.47
			N+	4.85 ± 0.18^***^	4.50 ± 0.51	81.08 ± 17.71	66.88 ± 11.46	65.43 ± 16.9
	PN/110R	Skin	N−	4.20 ± 0.71	5.72 ± 0.37	24.93 ± 2.93	25.94 ± 3.31	36.48 ± 22.22
			N+	7.28 ± 0.72^**^	5.03 ± 1.00	22.14 ± 3.41	24.3 ± 2.75	73.72 ± 25.99
		Pulp	N−	4.17 ± 2.03	4.78 ± 0.75	98.83 ± 24.72	81.29 ± 16.8	25.11 ± 6.14
			N+	6.02 ± 0.11	4.23 ± 0.45	69.42 ± 0.50	61.8 ± 3.55	64.86 ± 38.31

Raw values; asterisks indicate a significant statistical difference between N+ and N– treatments. Data shown are means ± standard deviation, n = 6. Statistical analyses have been done using a t-test, (

* P < 0.05;

** P < 0.01;

*** P < 0.001).

**Table:** 

**(B)**								
		**Analysis variance**	**Malate (mmoles/g DW)**	**Tartrate (mmoles/g DW)**	**Glucose (mmoles/g DW)**	**Fructose (mmoles/g DW)**	**Total AA (nmol/mg DW)**
CS	Skin	Years (Y)	^**^	NS	^*^	NS	^***^
		Treatment (T)	^***^	NS	NS	^***^	^***^
		Rootstock (R)	^*^	^*^	^***^	^***^	^*^
		Y/T	NS	NS	NS	NS	NS
		Y/R	NS	NS	NS	NS	NS
		T/R	NS	NS	NS	NS	NS
		Y/T/R	NS	NS	NS	NS	NS
	Pulp	Years (Y)	^**^	NS	NS	NS	^*^
		Treatment (T)	NS	NS	NS	NS	^**^
		Rootstock (R)	NS	^**^	NS	NS	NS
		Y/T	^**^	^**^	^*^	^*^	NS
		Y/R	NS	NS	NS	NS	NS
		T/R	NS	NS	NS	NS	NS
		Y/T/R	NS	NS	NS	NS	NS
PN	Skin	Years (Y)	^***^	NS	NS	NS	^**^
		Treatment (T)	^***^	NS	NS	NS	^*^
		Rootstock (R)	^***^	NS	NS	NS	NS
		Y/T	^***^	NS	NS	NS	NS
		Y/R	^*^	NS	NS	NS	NS
		T/R	NS	^**^	NS	NS	NS
		Y/T/R	NS	NS	NS	NS	NS
	Pulp	Years (Y)	^***^	NS	NS	NS	^**^
		Treatment (T)	^**^	NS	^*^	^*^	NS
		Rootstock (R)	^*^	^*^	NS	NS	NS
		Y/T	^*^	NS	NS	NS	NS
		Y/R	NS	NS	NS	NS	NS
		T/R	NS	^*^	^*^	NS	NS
		Y/T/R	NS	NS	NS	NS	NS

ANOVA analysis. Statistical analyses have been done using an analysis of variance with years (Y), rootstock (R), treatment (T), and their interaction effects (ns, P > 0.05;

* P < 0.05;

** P < 0.01;

*** P < 0.001). DW, dry weight.

### Effects of nitrogen supply on primary metabolites in the berries of different rootstock/scion combinations

Table 3 shows that for all samples, the sugar concentration was much higher in the pulp than in the skins, independently of the rootstock genotype and nitrogen supply. In CS, nitrogen supply did not affect the sugar content of the skin and of the pulp, while it decreased the sugar content of the pulp in PN berries.

ANOVA showed that the rootstock genotype significantly affected the skin content of organic acids, hexoses and amino acids in CS, while it only affected tartrate in CS pulp and malate in PN skin (Table 3A). For a given compartment (skin or pulp), and whatever the variety, the malate content was generally higher on 110R than on RGM, while the reverse was observed for tartrate (Table 3B).

Whatever the rootstock used, the malate content in CS was increased by higher nitrogen supply and this effect was also more marked in the skin than in the pulp. This effect of nitrogen on malate content was absent for PN.

Nitrogen supply significantly increased the total amino acid content of the skin and pulp of the berries for almost all rootstock/scion combinations. However, the effect was less marked in PN skin, and even absent in PN pulp (Table 3B).

For the CS/RGM combination, increasing nitrogen supply strongly increased the amino acid concentration both in the skin and in the pulp in 2013 and 2014, although this effect was less marked in 2014 (Table 3B). Malate content was increased by higher nitrogen supply in the skin for both years, but only in 2013 for the pulp. The sugar concentration was much higher in the pulp than in the skin, but was not significantly affected by nitrogen.

For the CS/110R combination, higher nitrogen supply also strongly enhanced the total amino acid concentration in the skin, but less in the pulp. The treatment also increased the skin malate content for both years. There was no consistent effect of nitrogen status on the sugar content.

For the PN/RGM combination, there was no consistent effect of nitrogen supply on the organic acids, and a marginal decrease of sugars in 2013 (Table 3B). The amino acid content was increased by higher nitrogen supply, especially in the skin in 2013.

For the PN/110R combination, there was no marked effect of nitrogen supply on malate, tartrate and sugars. While the total amino acid concentration was increased by high nitrogen supply both in the skin and in the pulp, the glucose and fructose concentrations were diminished in the pulp.

The skin was generally more reactive than the pulp to the different parameters studied, and among these parameters, the most sensitive were the malate and amino acid contents (Table 3A).

### Effects of nitrogen supply on the berries of different rootstock/scion combinations as assessed by untargeted metabolomics

In order to investigate the impact of rootstock and different nitrogen status on the metabolome of grape berries, an untargeted LC-ESI-MS approach was developed with two different cultivars, Cabernet Sauvignon (CS), and Pinot Noir (PN). Two different rootstock (RGM and 110R) and three different nitrogen conditions (limited, 0.8 mM, N−; regular, 1.4 mM, N–0; excessive, 3.6 mM, N+) were used. Skin and pulp were analyzed separately.

The analysis in negative ionization mode allowed to detect secondary metabolites that mainly belong to the groups of anthocyanins, flavonols, flavan-3-ols/procyanidins, stilbenes, hydroxycinnamic, and hydroxybenzoic acids. The m/z values, retention time and putative identification of the detected molecules are reported in Supplementary File 1.

Since LC-MS based metabolomics is prone to effects such as matrix effect and ion suppression/enhancement that can impair the comparison between samples, we checked the performance of our analytical platform for relative quantitation. We compared the results of LC-MS determination with those obtained with HPLC-DAD, which is a fully quantitative technique. Nine samples randomly selected, with all their replicates, were analyzed by HPLC-DAD and compared with HPLC-MS. As shown in Supplementary Figure 1, the HPLC-DAD and HPLC-MS anthocyanin relative quantitation was fairly comparable. The performance of our metabolomics platform on grape berry extracts has been extensively discussed in a previous paper (Toffali et al., 2011).

**Figure 1:**
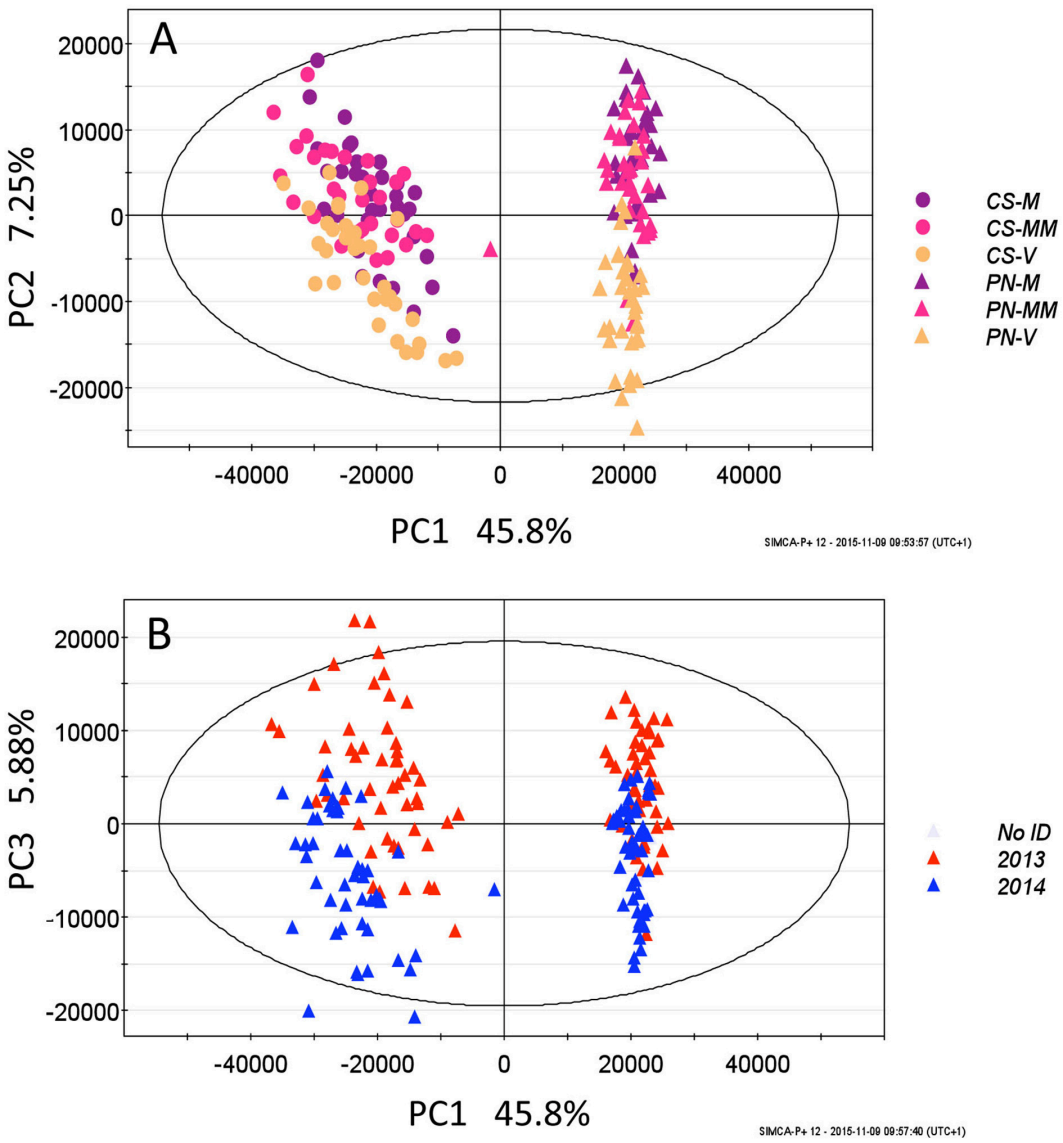
**PCA score plot of skin datamatrix, PC (Principal component)1-PC2 (A) and PC1-PC3 (B), in which the metabolites are responsible of the clustering of the two cultivars (A), the ripening stages (veraison, mid-maturity and maturity) (A) and the two vintages (B)**. CS, Cabernet-Sauvignon; PN, Pinot noir; V, veraison; MM, mid maturity; M, maturity.

As a first approach, the two LC-MS datamatrix (pulp, skin) were preliminary explored by the unsupervised Principal Component Analysis (PCA). This analysis on skin datamatrix, showed, as expected, that the samples group primarily according to the cultivars (Figure 1A), then according to the ripening stage (Figure 1A), and finally according to the vintage (Figure 1B). Thus, separate Orthogonal Bidirectional Partial Least Square Discriminant Analysis (O2PLS-DA) models were built to highlight the main differences between the two cultivars, the three ripening stages and the two vintages. PN and CS berries differed mainly for anthocyanin accumulation, more abundant in Cabernet Sauvignon, and for the different flavonoid distribution, with flavonols more abundant in CS and flavanones/flavanols more abundant in PN (Supplementary Figure 2). As minor difference, resveratrol is higher in mature Pinot noir grape berries. In terms of ripening, increases of resveratrol/stilbenes and significant changes in anthocyanins and flavonoid profiles were observed in both cultivars (Supplementary Figures 2, 3). The pulp datamatrix showed that CS contains much more tryptophan N-glucoside and less hydroxybenzoic acids than PN, and that both these metabolites increase during ripening (Supplementary Figures 2E–G).

**Figure 2:**
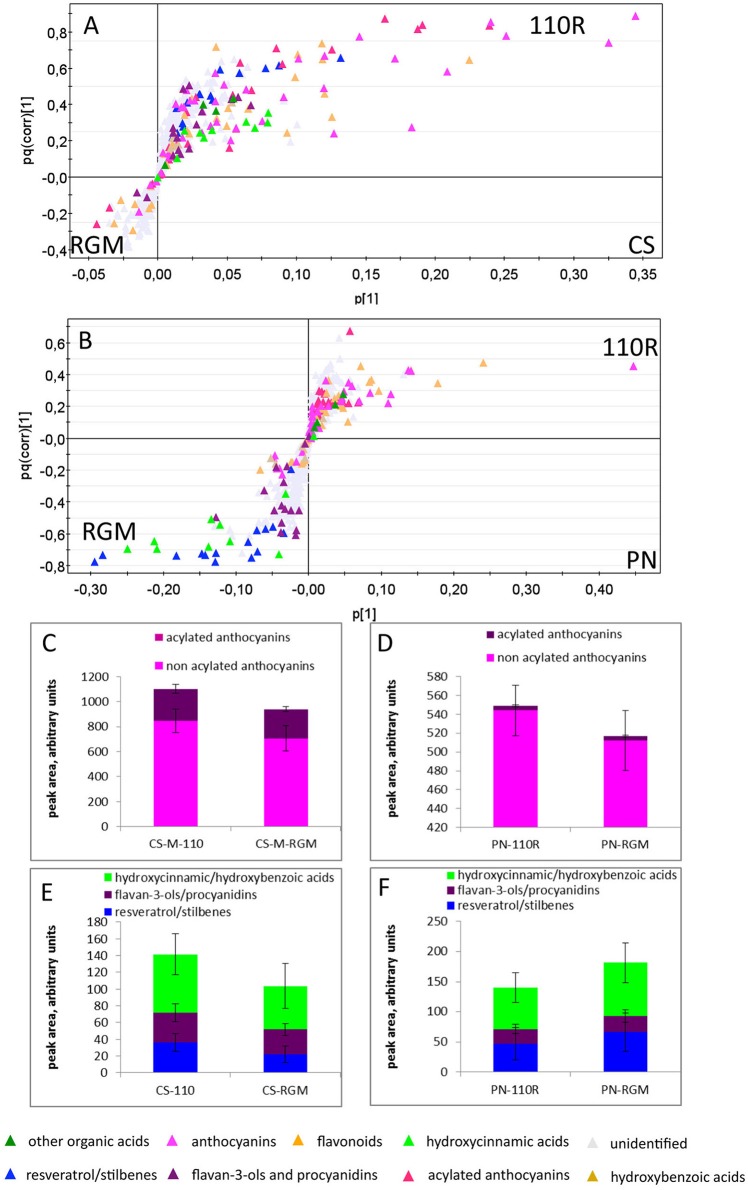
**O2PLS-DA loading plot (A,B) showing the CS-110R, PN-110R, CS-RGM, PN-RGM cultivar-rootstock consortia clustered according to the grape berries skin metabolites; in C–F the average relative levels of anthocyanins, hydroxycinnamic acids, flavan-3-ols/procyanidins and resveratrol/stilbenes of the four consortia are shown, ± standard deviation**. The high standard deviations of these data is expected and depend on their mixed nature, since for each consortia data of two ripening stages (M, MM), two vintages (2013 and 2014) and three nitrogen nutrition levels are clustered. CS, Cabernet-Sauvignon; PN, Pinot noir; 110R, 110R rootstock; RGM, RGM rootstock.

The two cultivars were then analyzed separately in more details, using the above supervised approach, and considering only the more mature stages (mid mature and mature) and the skin datamatrix. Both the cultivars showed rootstock-dependent differences (Figure 2): the CS-110R combination was more advantageous for secondary metabolites, especially anthocyanins, hydroxycinnamic acids, resveratrol/stilbenes and flavan-3-ols/procyanidins, compared with CS-RGM (Figures 2A,C,E). Also the combination PN-110R accumulated higher levels of anthocyanins, while the PN-RGM combination accumulated higher levels of hydroxycinnamic acids, flavan-3-ols/procyanidins, resveratrol/stilbenes (Figures 2B,D,F).

**Figure 3:**
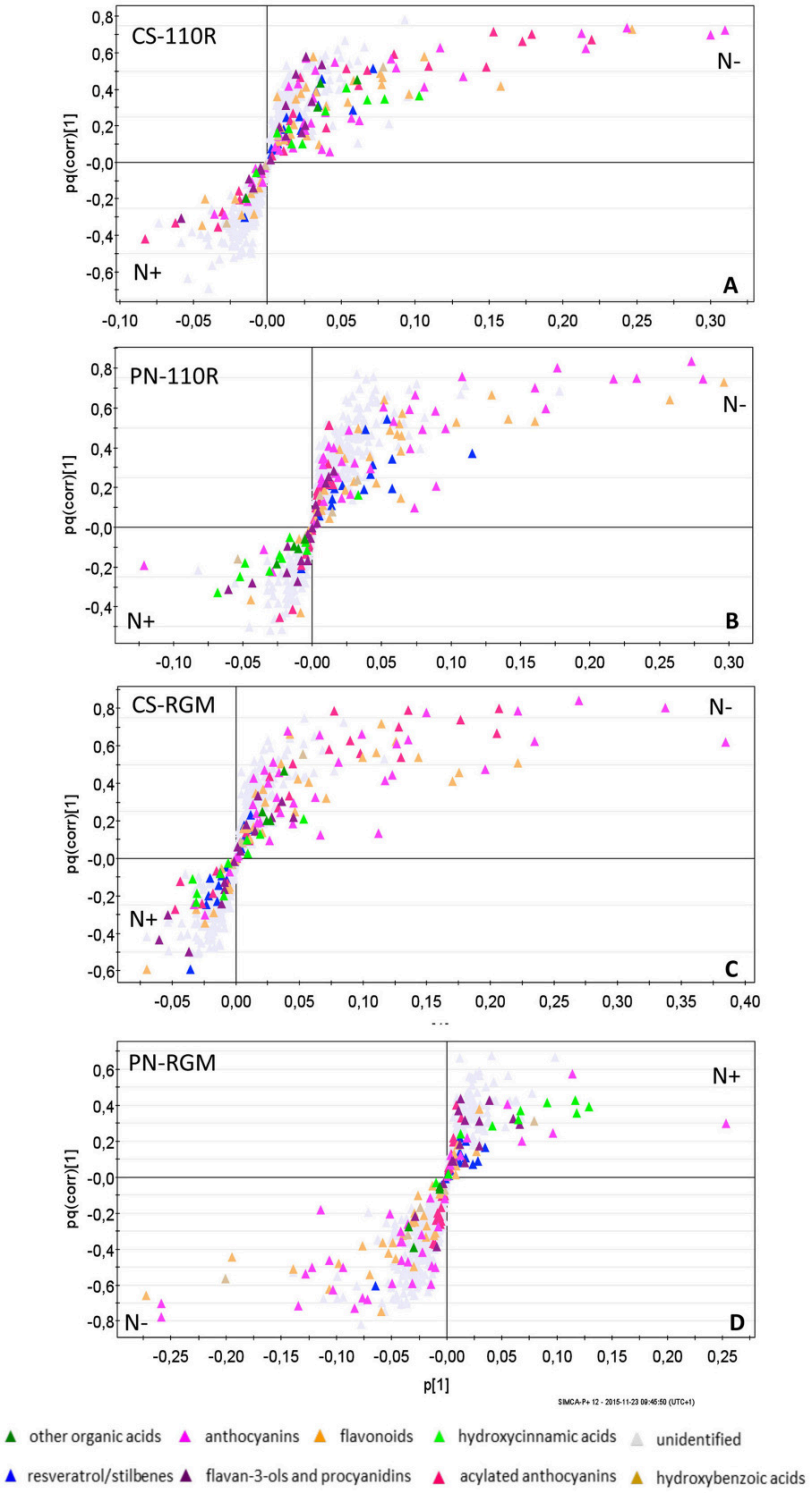
**O2PLS-DA loading plot showing the CS-110R, PN-110R, CS-RGM, PN-RGM samples in two nitrogen nutrition status (*N*– = 0.8 mM; *N*+ = 3.6 mM) clustered according to their skin grape berry metabolites**. CS, Cabernet-Sauvignon; PN, Pinot noir; 110R, 110R rootstock; RGM, RGM rootstock.

The four combinations (CS-110R, CS-RGM, PN-110R, and PN-RGM) were then analyzed under the different nitrogen nutrition conditions. The supervised O2PLS-DA multivariate analysis resulted in very weak models when the three different nitrogen supplementations were separately considered; the exclusion of the intermediate nitrogen supply resulted in stronger models. Thus, the effect of limited nitrogen supply was compared with higher nitrogen (Figure 3). In all four combinations between the two cultivars and the two rootstocks, excessive nitrogen supply decreased the accumulation of flavonoids and anthocyanins (Figure 3).

These effects were higher in PN than in Cabernet Sauvignon; in CS cultivar only cyanidin, delphinidin, and petunidin-based anthocyanins were affected by different levels of nitrogen nutrition, while in PN the peonidin-based anthocyanins were also affected.

The inhibition of accumulation of secondary metabolites caused by high nitrogen supplementation was higher when the scions were grafted on the 110R rootstock, compared with RGM (Supplementary Figure 4).

## Discussion

The berry content at harvest, which determines the quality of table grapes and is a major determinant for wines depend on complex interactions between the rootstocks, the scion, and their respective environment (soil and atmosphere) that may be modified by a wide range of viticultural practices. To reach a precise control of berry content, and to face more easily current challenges like those raised by climate change, it is important to document and understand the different levers offered by the manipulation of rootstock/scion/environment interactions. The present study investigates the interactions between nitrogen level/rootstock genotype and scion genotype with targeted analytical procedures and an untargeted metabolomic approach.

A high vigor rootstock grafted by a scion Chardonnay (Ough et al., 1968) or Merlot (Stockert et al., 2013) tend to acquire more N, resulting in higher amounts of must amino-N than the intermediate and low growth promoting rootstocks. Our results (Table XX) partially confirm and precise this conclusion by comparing the skin and the pulp responses. The amino acid concentration was affected by the rootstock in the skin of CS, but not in the pulp, while the rootstock did not affect the amino acid content of PN berries.

Although nitrogen fertilization did not significantly affect berry size and impacted only marginally secondary leaf development, it significantly impacted the malate and amino acid content of berries for almost all rootstock/scion combinations. This effect of nitrogen on amino acids has already been described for other rootstock/scion combinations (Holzapfel and Treeby, 2007). The effects of nitrogen on malate are less expected. Our work also shows differential effects on a given treatment on the skin and pulp compartment. The increase in malate induced by high nitrogen is more marked in CS skin and pulp. The rootstock genotype only affects tartrate in CS pulp and malate in PN skin. This underlines the complexity of the soil composition/roostock/scion interactions, which may be selective for one variety, one rootstock, one compound and one berry compartment, and depend on year (climate). The analysis of primary metabolites suggest that the skin was the more reactive compartment, and this compartment was further analyzed by untargeted metabolomics.

This allowed to detect secondary metabolites belonging to the groups of anthocyanins, flavonols, flavan-3-ols/procyanidins, stilbenes, hydroxycinnamic, and hydroxybenzoic acids. A number of peaks are still unidentified. The samples separated according to varieties, ripening stage and vintage. In CS berries, the RGM rootstock favored a higher amount of anthocyanins, hydroxycinnamic acids, resveratrol/stilbenes and flavan-3-ols/procyanidins than the 110R rootstock.

Whatever the rootstock/scion combination, high nitrogen decreased the amounts of flavonoids and anthocyanins. This is in agreement with Soubeyrand et al. (2014) who found that low nitrogen supply significantly increased the anthocyanin level in Cabernet Sauvignon berries collected from field plants at two ripening stages (26 days post-véraison and maturity).

The present study show that these effects of nitrogen are variety-dependent, and do not concern all anthocyanins. They were stronger in PN than in CS. Only cyanidin, delphinidin, and petunidin-based anthocyanins were affected by nitrogen in CS berries, while the peonidin-based anthocyanins were also affected in PN berries.

Finally, the amounts of secondary metabolites caused by high nitrogen supply were more decreased in berries collected on scions grafted on 110R rootstock than on RGM (Supplementary Figure 4). As the present experiments were conducted in pots limiting rootstock development and as the vines were pruned similarly, the differences observed might be related more to a higher intrinsic capacity to retrieve and transport nitrogen for 110 R compared RGM than to vigor (vegetative development) *per-se*. Indeed, Lecourt et al. (2015) have shown that response to nitrate supply in grafted grapevines alter the root and shoot distribution of various ions in a genotype dependent way. Our former work (Berdeja et al., 2014) also showed that the rootstock genotype (110R, high vigor or 125 AA, low vigor) significantly impacted the total amount of anthocyanins of PN berries grown in field conditions. The proportion of 3′, 4′–dihydroxy cyanidin and peonidin and 3′, 4′, 5′–trihydroxy delphinidin, malvidin, and petunidin slightly varied depending on the year, but was not clearly modified by rootstock or water supply.

The data described here show that untargeted metabolomics may be a powerful technique to detect the numerous and subtles changes depending on soil composition/rootstock/scion/climate interactions. The build up of adequate data bases and the combination of these data with RNAseq approaches would be very useful to decipher the overall response of berry metabolism, and the underlying gene expression changes to environmental cues and genetic background.

## Author contributions

CV and SD designed and oversaw the research. AH, GH, and PH performed the field experiments and berry sampling; AH, GH, SN, and MC did the metabolic and metabolomic analysis; AH and MC analyzed data. FG and SD drafted the ms. EG and NO critically revised the manuscript. All authors read and approved the final manuscript.

### Conflict of interest statement

The authors declare that the research was conducted in the absence of any commercial or financial relationships that could be construed as a potential conflict of interest. The handling Editor declared a shared affiliation, though no other collaboration, with several of the authors MC, SN, FG and states that the process nevertheless met the standards of a fair and objective review.

## Supplementary material

Supplementary Figure 1 **Comparison between relative quantitation performances of HPLC-ESI-MS and HPLC-DAD: heat map representing the relative levels of each of the HPLC-DAD-detectable metabolites in a set of randomly selected samples, as detected by HPLC-DAD and HPLC-ESI-MS**. DI, delphinidin; Cy, cyanidin; Pt, petunidin; Peo, peonidin; MV, malvidin; hx, hexose; ac, acetyl; cou, coumaroyl; 110R, 110R rootstock; RGM, RGM rootstock; V, veraison; MM, mid-maturity; M, maturity; N-: 0.8 mM nitrogen supply; N-0: 1.4 mM nitrogen supply; N+: 3.6 mM nitrogen supply.

Supplementary Figure 2 **Differences between Cabernet Sauvignon and Pinot noir cultivars grape berries explored by LC-MS-based untargeted metabolomics of skin (A–D) and pulp (E–G) grape berry: (A) and (E), O2PLS-DA loading plot**. The high standard deviations of the average relative level of metabolite data is expected and depend on their mixed nature, since for each cultivar data of two ripening stages (M, MM), two vintages (2013 and 2014), two rootstock (110R ans RGM) and three nitrogen nutrition levels are clustered. CS, Cabernet-Sauvignon; PN, Pinot noir; V, veraison; MM, mid mature; M, mature.

Supplementary Figure 3 **Evolution of grape berry ripening in Cabernet sauvignon (A,B) and Pinot noir (C,D) explored by LC-MS-based untargeted metabolomics of grape berry skin; (A,C): O2PLS-DA score plot; (B,D): O2PLS-DA loading plot**. The high standard deviations of the average relative level of metabolite data is expected and depend on their mixed nature, since for each cultivar data for two rootstocks (110R and RGM) and three nitrogen nutrition levels are clustered. CS, Cabernet-Sauvignon; PN, Pinot noir; V, veraison; M, maturity.

Supplementary Figure 4 **Average relative level of metabolite in the grape berry skin of CS-110R, PN-110R, CS-RGM, PN-RGM cultivar-rootstock consortia in two conditions of nitrogen nutrition, +/− the standard deviation**. The high standard deviations of the average relative level of metabolite data is expected and depend on their mixed nature, since for each cultivar data two ripening stages (mid maturity, maturity) and two vintages (2013, 2014) are clustered. CS, Cabernet-Sauvignon; PN, Pinot noir; 110R, 110R rootstock; RGM, RGM rootstock; N-: 0.8 mM nitrogen supply; N+: 3.6 mM nitrogen supply.

Supplementary File 1 **List of metabolites, isotopes, adducts, putatively identified by HPLC-ESI-MS in grape berries skin and pulp**.

Supplementary Table 1 **Measurements of primary and secondary leaf area at harvest for the different rootstock/scion combinations fertilized by 0.8 mM (N–) or 3.6 mM (N+)**. Data shown are means ± standard deviation, *n* = 6. Statistical analyses were done using an analysis of variance with years (Y), rootstock (R), treatment (T), variety (V), and their interaction effects (ns, *P* > 0.05; ^*^*P* < 0.05; ^**^*P* < 0.01; ^***^*P* < 0.001).

Supplementary Table 2 **Measurements of berry weight at harvest for the different rootstock/scion combinations fertilized by 0.8 mM (N–) or 3.6 mM (N+). (A)** The data are means ± standard deviation, *n* = 6. **(B)** Statistical analyses have been done using an analysis of variance with years (Y), rootstock (R), treatment (T), variety (V), and their interaction effects (ns, *P* > 0.05; ^*^*P* < 0.05; ^**^*P* < 0.01; ^***^*P* < 0.001).

Supplementary Table 3 **Petiole total N content as affected by nitrogen supply**. Values are means of 3 independent replicates + SE. N− : 0.8 mM N ; N+ : 3.6 mM N. One factor (N treatment) Anova tests were made, as well as Tukey tests. For each rootstock/variety combination, a and b indicate significantly different values between N− and N+ treatment. . ‡ Unique value. Statistical analyses were done using an analysis of variance with years (Y), rootstock (R), treatment (T), variety (V) and their interaction effects (ns, *P* > 0.05; ^*^*P* < 0.05; ^**^*P* < 0.01; ^***^*P* < 0.001).

Supplementary Table 4 **Effect of nitrogen supply on yeast assimilable nitrogen (mg/L) in musts prepared from different rootstocks/scion combinations**. In 2014, values are means of 3 independent replicates + SE ± Unique value were made in 2013; N–: 0.8 mM N; N+: 3.6 mM N. In 2014, one factor (N treatment) Anova tests were made. For each rootstock/variety combination, a, b, and c indicate significantly different values between the means.

## Abbreviations

CS: Cabernet Sauvignon
DW: dry weight
ESI: electrospray ionization source
HPLC: High Pressure Liquid Chromatography
N: nitrogen
PN: Pinot Noir
LC-DAD: Liquid chromatography-diode array detector)
LC-MS: Liquid chromatography-mass spectrometry
PCA: Principal Component Analysis
OPLS-DA: supervised Orthogonal Partial Least Square Discriminant Analysis
O2PLS-DA: Orthogonal Bidirectional Partial Least Square Discriminant Analysis
RGM: rootstock Riparia Gloire de Montpellier
110 R: rootstock Richter.
