# Supplementary material for: Roostocks/Scion/Nitrogen Interactions Affect Secondary Metabolism in the Grape Berry
Source: Front Plant Sci. 2016 Aug 9;7:1134. doi: 10.3389/fpls.2016.01134 (PMC4977291; doi:10.3389/fpls.2016.01134)

**Supplementary Table 1.**

| **Years** | **Scion/rootstock**  **combination** | **Nitrate supply** | **Primary leaf area (m²)** | **Secondary leaf area (m²)** |
| --- | --- | --- | --- | --- |
| **2013** | CS/RGM | N- | 0.68 ± 0.14 | 0.40 ± 0.20 |
|  |  | N+ | 0.72 ± 0.28 | 0.53 ± 0.11 |
|  | CS/110R | N- | 0.64 ± 0.16 | 0.34 ± 0.09 |
|  |  | N+ | 0.97 ± 0.19 | 0.50 ± 0.19 |
|  | PN/RGM | N- | 0.90 ± 0.23 | 0.34 ± 0.14 |
|  |  | N+ | 0.98 ± 0.08 | 0.58 ± 0.09 |
|  | PN/110R | N- | 0.86 ± 0.14 | 0.39 ± 0.09 |
|  |  | N+ | 0.89 ± 0.11 | 0.42 ± 0.06 |
| **2014** | CS/RGM | N- | 0.86 ± 0.23 | 0.19 ± 0.07 |
|  |  | N+ | 0.95 ± 0.14 | 0.63 ± 0.05 |
|  | CS/110R | N- | 0.77 ± 0.06 | 0.32 ± 0.11 |
|  |  | N+ | 0.73 ± 0.28 | 0.68 ± 0.13 |
|  | PN/RGM | N- | 1.71 ± 0.11 | 0.33 ± 0.01 |
|  |  | N+ | 1.47 ± 0.72 | 0.63 ± 0.12 |
|  | PN/110R | N- | 1.48 ± 0.53 | 0.22 ± 0.09 |
|  |  | N+ | 2.47 ± 0.73 | 0.57 ± 0.11 |

| **Analysis of variance** | **Primary leaf area** | **Secondary leaf area** |
| --- | --- | --- |
| Years (Y) | *** | *** |
| Treatment (T) | NS | *** |
| Variety (V) | *** | *** |
| Rootstock (R) | NS | NS |
| Y/T | NS | NS |
| Y/V | *** | *** |
| T/V | NS | ** |
| Y/R | NS | NS |
| T/R | NS | NS |
| Y/T/V | NS | ** |
| Y/T/R | NS | NS |
| Y/V/R | NS | NS |
| T/V/R | NS | NS |
| Y/T/V/R | NS | NS |

A B

**Supplementary Table 2.**

A B

| Years | Scion/rootstock  combination | Nitrate supply | 100 berry weight (g) |
| --- | --- | --- | --- |
| 2013 | CS/RGM | N- | 1.21 ± 0.09 |
|  |  | N+ | 1.12 ± 0.01 |
|  | CS/110R | N- | 1.04 ± 0.03 |
|  |  | N+ | 1.09 ± 0.15 |
|  | PN/RGM | N- | 1.48 ± 0.12 |
|  |  | N+ | 1.46 ± 0.06 |
|  | PN/110R | N- | 1.24 ± 0.08 |
|  |  | N+ | 1.36 ± 0.07 |
| 2014 | CS/RGM | N- | 1.39 ± 0.04 |
|  |  | N+ | 1.37 ± 0.06 |
|  | CS/110R | N- | 1.25 ± 0.05 |
|  |  | N+ | 1.22 ± 0.06 |
|  | PN/RGM | N- | 1.43 ± 0.17 |
|  |  | N+ | 1.42 ± 0.10 |
|  | PN/110R | N- | 1.45 ± 0.20 |
|  |  | N+ | 1.30 ± 0.05 |

| **Analysis of variance** | 100 berry weight |
| --- | --- |
| Years (Y) | ** |
| Treatment (T) | NS |
| Variety (V) | *** |
| Rootstock (R) | *** |
| Y/T | NS |
| Y/V | ** |
| T/V | NS |
| Y/R | NS |
| T/R | NS |
| Y/T/V | NS |
| Y/T/R | * |
| Y/V/R | NS |
| T/V/R | NS |
| Y/T/V/R | NS |

**Supplementary figure 1**


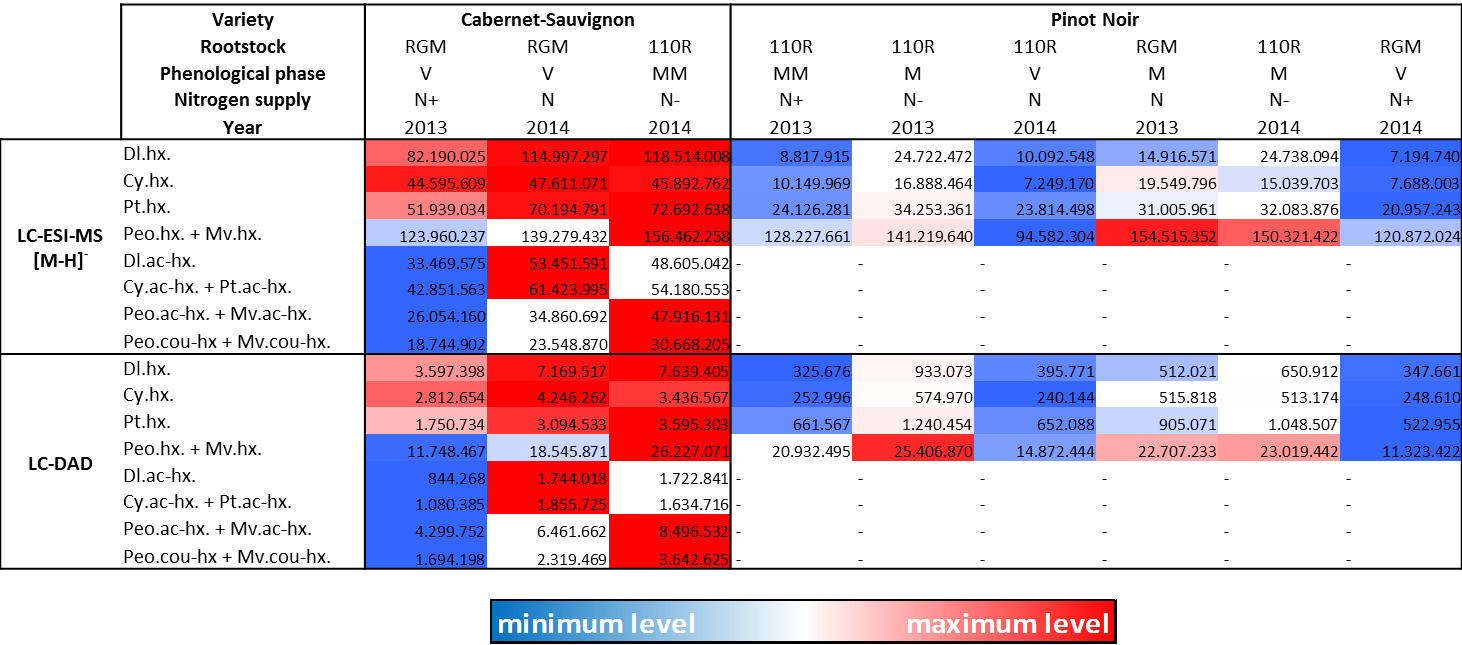


**Supplementary figure 2**


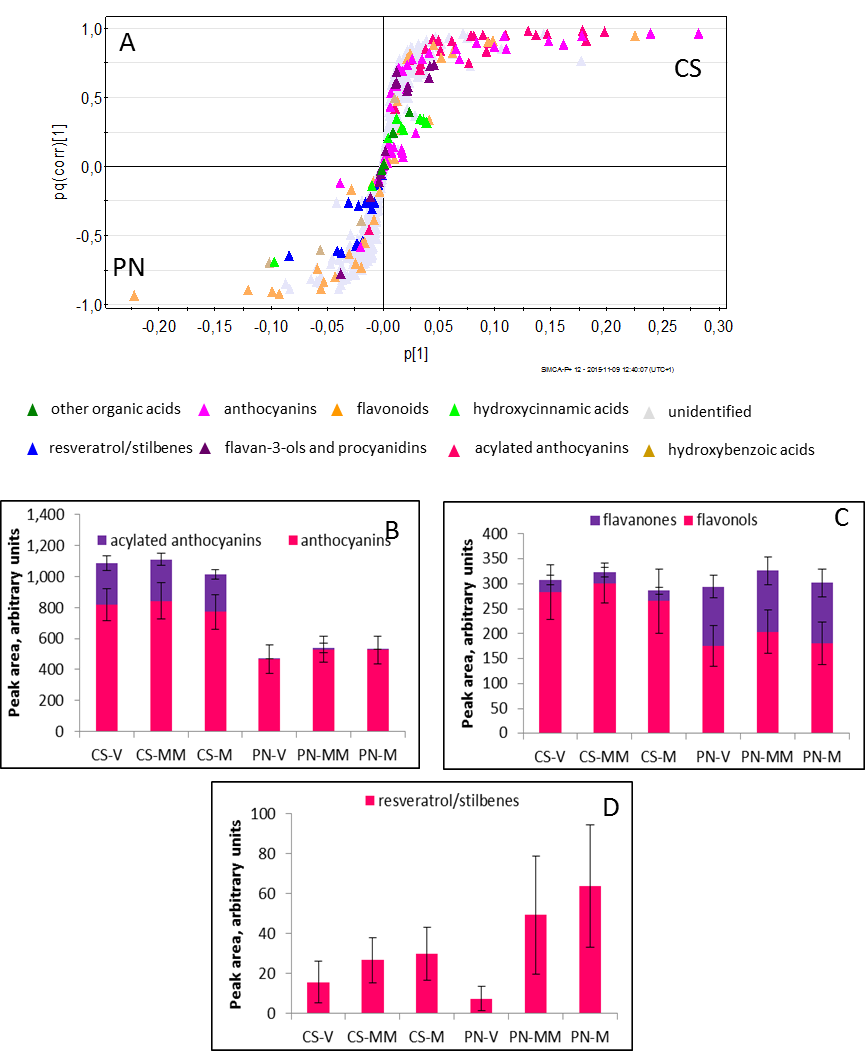


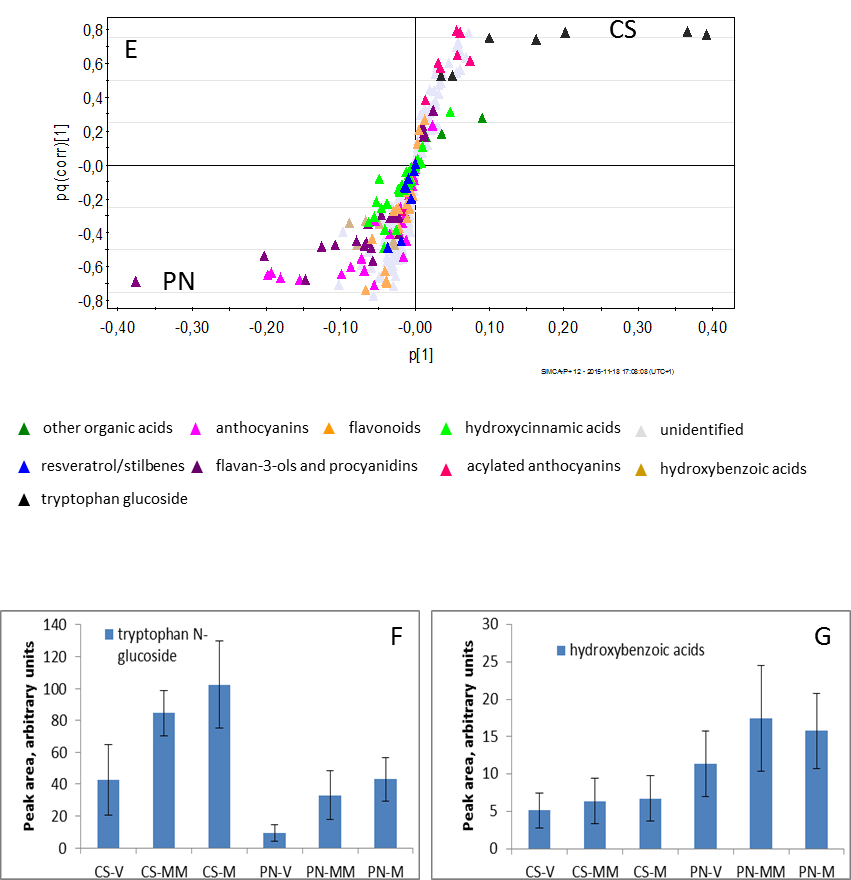


**Supplementary figure 3**


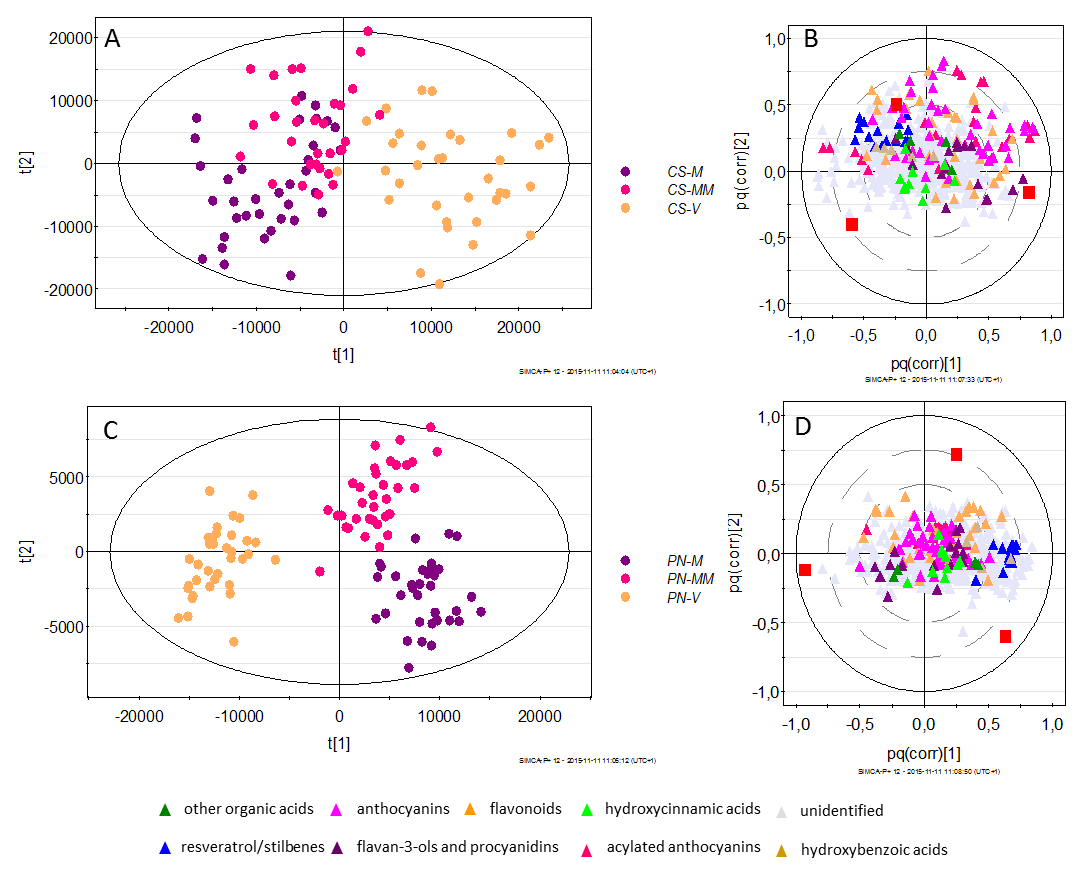


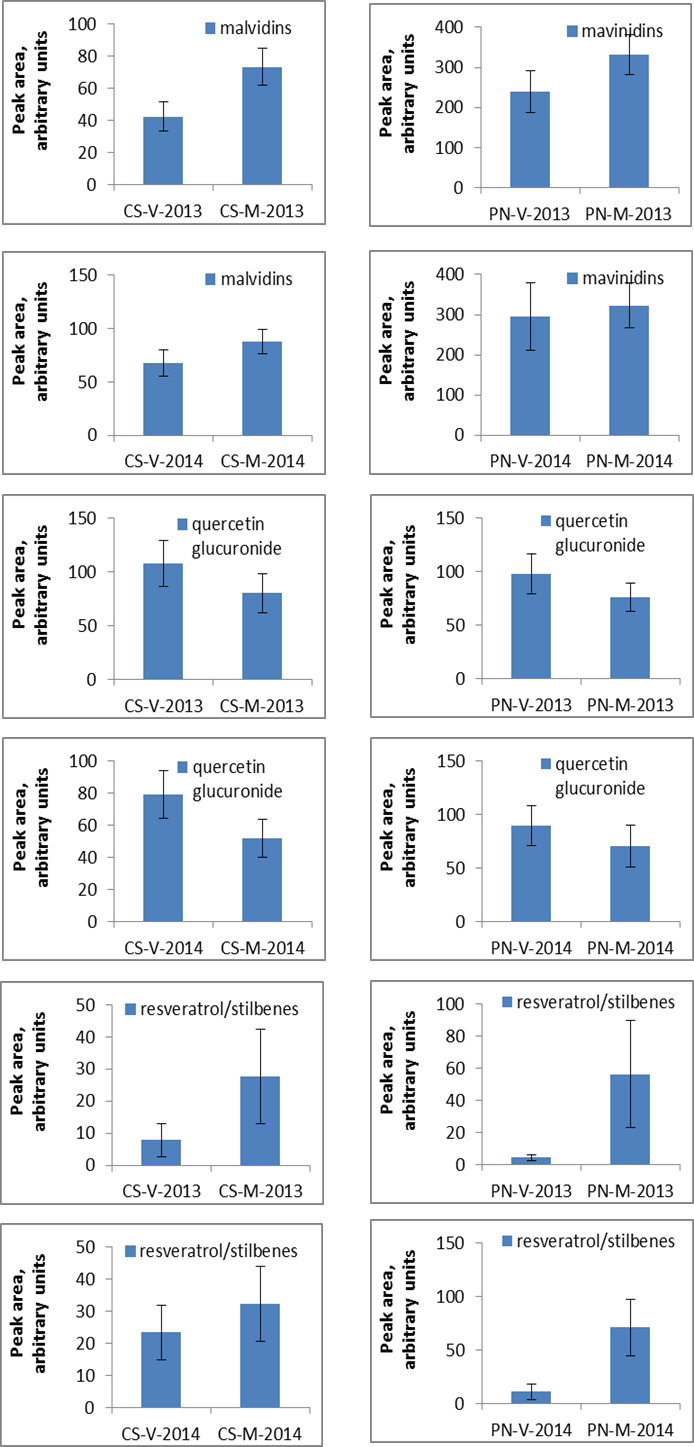


**Supplementary figure 4**


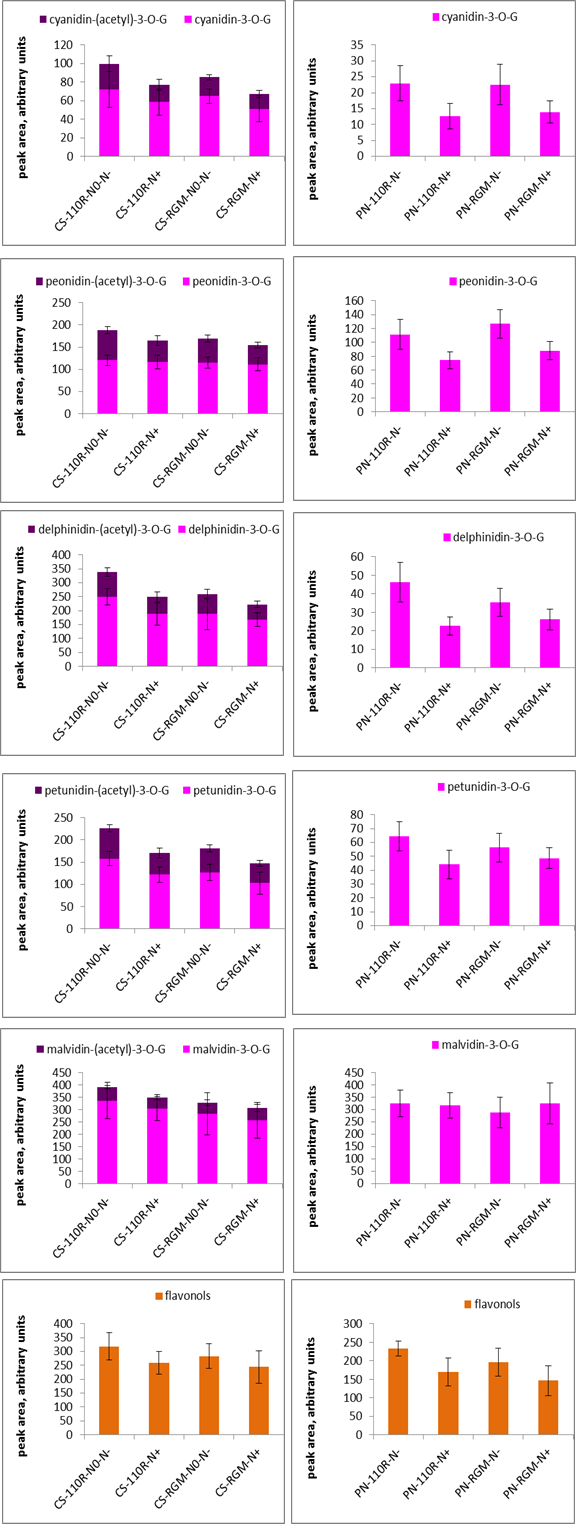

Supplement: Supplementary Figure 1 — Comparison between relative quantitation performances of HPLC-ESI-MS and HPLC-DAD: heat map representing the relative levels of each of the HPLC-DAD-detectable metabolites in a set of randomly selected samples, as detected by HPLC-DAD and HPLC-ESI-MS. DI, delphinidin; Cy, cyanidin; Pt, petunidin; Peo, peonidin; MV, malvidin; hx, hexose; ac, acetyl; cou, coumaroyl; 110R, 110R rootstock; RGM, RGM rootstock; V, veraison; MM, mid-maturity; M, maturity; N-: 0.8 mM nitrogen supply; N-0: 1.4 mM nitrogen supply; N+: 3.6 mM nitrogen supply. [file DataSheet1.DOCX]
