# Supplementary material for: Roostocks/Scion/Nitrogen Interactions Affect Secondary Metabolism in the Grape Berry
Source: Front Plant Sci. 2016 Aug 9;7:1134. doi: 10.3389/fpls.2016.01134 (PMC4977291; doi:10.3389/fpls.2016.01134)
Supplement: Supplementary Table 3 — Petiole total N content as affected by nitrogen supply. Values are means of 3 independent replicates + SE. N− : 0.8 mM N ; N+ : 3.6 mM N. One factor (N treatment) Anova tests were made, as well as Tukey tests. For each rootstock/variety combination, a and b indicate significantly different values between N− and N+ treatment. . ‡ Unique value. Statistical analyses were done using an analysis of variance with years (Y), rootstock (R), treatment (T), variety (V) and their interaction effects (ns, P > 0.05; *P < 0.05; **P < 0.01; ***P < 0.001). [file Table3.pdf]

Supplementary Table 3. Petiole total N content as affected by nitrogen supply. Values are means of 3 independent replicates + SE. N- : 0.8 mM N ; N+ : 3.6 mM N. One factor (N treatment) Anova tests were made, as well as Tukey tests. For each rootstock/variety combination, a and b indicate significantly different values between N- and N+ treatment. . [ǂ]Unique value. Statistical analyses were done using an analysis of variance with years (Y), rootstock (R), treatment (T), variety (V) and their interaction effects (ns, $P > 0.05$; *, $P < 0.05$; **, $P < 0.01$; ***, $P < 0.001$).

	**Cabernet Sauvignon**		**Pinot Noir**	
	**RGM**	**110R**	**RGM**	**110R**
**2013**				
N-	0.23 ± 0.01 a	0.25 ± 0.05 a	0.28 [ǂ]	0.21 [ǂ]
N	0.31 ± 0.02 a	0.31 ± 0.05 a	0.30 [ǂ]	0.21 [ǂ]
N+	0.37 ± 0.12 a	0.48 ± 0.18 a	0.37 [ǂ]	0.37 [ǂ]
**2014**				
N-	0.38 ± 0.10 a	0.39 ± 0.04 a	0.33 ± 0.01 a	0.30 ± 0.02 a
N	0.62 ± 0.02 b	0.47 ± 0.04 ab	0.35 ± 0.03 a	0.37 ± 0.01 a
N+	0.66 ± 0.10 b	0.55 ± 0.12 b	0.63 ± 0.12 b	0.60 ± 0.08 b

	**Analysis of variance**		
		**CS**	**PN**
Variety	NS	-	-
Year	NS	NS	-
Rootstock	NS	NS	NS
