# Supplementary material for: Roostocks/Scion/Nitrogen Interactions Affect Secondary Metabolism in the Grape Berry
Source: Front Plant Sci. 2016 Aug 9;7:1134. doi: 10.3389/fpls.2016.01134 (PMC4977291; doi:10.3389/fpls.2016.01134)
Supplement: Supplementary Table 4 — Effect of nitrogen supply on yeast assimilable nitrogen (mg/L) in musts prepared from different rootstocks/scion combinations. In 2014, values are means of 3 independent replicates + SE ± Unique value were made in 2013; N–: 0.8 mM N; N+: 3.6 mM N. In 2014, one factor (N treatment) Anova tests were made. For each rootstock/variety combination, a, b, and c indicate significantly different values between the means. [file Table4.pdf]

Supplementary Table 4. Effect of nitrogen supply on yeast assimilable nitrogen (mg/L) in musts prepared from different rootstocks/scion combinations. In 2014, values are means of 3 independent replicates + SE. ‡Unique value were made in 2013 ; N- : 0.8 mM N ; N+ : 3.6 mM N. In 2014, one factor (N treatment) Anova tests were made. For each rootstock/variety combination, a, b and c indicate significantly different values between the means.

	**Cabernet Sauvignon**		**Pinot Noir**	
	**RGM**	**110R**	**RGM**	**110R**
**2013**				
N-	120.00‡	180.00‡	180.00‡	164.00‡
N	147.00‡	255.00‡	187.00‡	180.00‡
N+	241.00‡	252.00‡	215.00‡	223.00‡
**2014**				
N-	252.67 ± 45.79 a	295.67 ± 30.83 a	268.00 ± 07.21 a	260.33 ± 47.35 a
N	283.00 ± 24.98 b	280.00 ± 24.64 a	233.00 ± 14.18 a	214.33 ± 15.57 a
**N+**	410.33 ± 11.68 c	490.00 ± 26.00 b	518.00 ± 25.87 b	568.00 ± 47.29 b
